# High School Basketball Coach and Player Perspectives on Warm-Up Routines and Lower Extremity Injuries

**DOI:** 10.1186/s40798-021-00328-4

**Published:** 2021-05-21

**Authors:** Corrine Munoz-Plaza, Dana Pounds, Anna Davis, Stacy Park, Robert Sallis, Manuel G. Romero, Adam L. Sharp

**Affiliations:** 1 Department of Research and Evaluation, Kaiser Permanente Southern California, 100 S. Los Robles Ave, 2nd Floor, Pasadena, CA 91101 USA; 2 Center for Effectiveness and Safety Research, Kaiser Permanente, Oakland, USA; 3 Department of Health System Science, Kaiser Permanente School of Medicine, Pasadena, USA; 4 Department of Family Medicine, Kaiser Permanente Medical Center, Fontana, CA USA; 5 University of the Pacific, Stockton, CA USA

**Keywords:** Athletic injuries, Team sports, Warm-up exercise, Qualitative research, Implementation science

## Abstract

**Background:**

While participation in sports-related activities results in improved health outcomes, high school athletes are at risk for lower extremity injuries, especially ankle, knee, and thigh injuries. Efforts to promote the adoption and implementation of evidence-driven approaches to reduce injury risk among school-aged athletes are needed. However, there is limited research regarding the perceived barriers, facilitators, and adherence factors that may influence the successful implementation of effective warm-up routines among this population.

**Methods:**

We conducted a qualitative study using semi-structured interviews and focus groups to assess high school basketball coach and player current practices, knowledge, and perspectives about warm-ups and lower-extremity injuries (LEIs). We interviewed coaches (*n* = 12) and players (*n* = 30) from May to October 2019. Participants were recruited from public high schools in a joint school district in Southern California. Multiple coders employed thematic analysis of the data using validated methods.

**Results:**

Coaches and players reported regular engagement (e.g., daily) in warm-up routines, but the time dedicated (range 5–45 min), types of exercises, and order varied substantially. Players often co-lead the warm-up practice with the coach or assistant coach. Despite regular engagement in warm-up, players and coaches report multiple challenges, including (1) limited time and space to warm-up effectively at games, (2) a perception that young players are not prone to injury, (3) competing demands for coaches’ time during practice, and (4) coaches’ lack of knowledge. Coaches and players perceive that warming up before practice will result in fewer injuries, and many players are motivated to warm up as a result of their personal injury experience; however, they desire guidance on the ideal exercises for preventing injury and training on the proper form for each exercise.

**Conclusions:**

Regular involvement in basketball warm-up routines is common among high school teams, but the methods and time dedicated to these practices varied. Players and coaches are eager for more information on warm-up programs shown to reduce LEIs.

**Supplementary Information:**

The online version contains supplementary material available at 10.1186/s40798-021-00328-4.

## Key Points

- We found high school basketball teams routinely warm up prior to practice but report barriers prior to games and lack knowledge and training about evidenced-based warm-up activities.
- Despite identified barriers to adherence, players and coaches are eager for more information on warm-up programs proven to reduce lower extremity injuries, as well as recommendations for how to effectively structure and sequence warm-up routines for maximum injury prevention.
- Further research is needed to better assess how barriers to warm-up may impact compliance with effective warm-up practices, since player and coach adherence is a key determinant of reaching desired outcomes to reduce lower extremity injuries.

## Introduction

Youth sports participation has positive benefits for young athletes, including increased physical, psychological, and social health [1, 2]. Studies have shown that involvement in sports and moderate-to-vigorous physical activity improves self-esteem, social skills, and personal well-being, while decreasing depressive symptoms, chronic diseases, and risky behaviors [2–4]. Despite these benefits, high school athletes are at risk for lower extremity injuries, especially ankle, knee, and thigh injuries [5, 6]. The National Federation of State High School Associations reports that approximately 1 million high school students participate in basketball every year [7]. Among high school players, about a quarter (23%) experience injuries with the highest rate of ankle/foot injuries occurring among boy (39.3%) and girl (36.4%) basketball players [8]. Given the benefits of athletic participation and these substantial rates of sports-related injuries, there is a need for the general adoption and implementation of evidence-driven approaches to reduce injury risk [9].

Structured neuromuscular warm-up routines have been shown to reduce lower extremity injury rates in youth athletes when applied consistently and with a high degree of compliance [10, 11]. Although injury prevention warm-up routines for youth are available, basketball-specific programs are lacking [12]. While some studies focusing on warm-up-based injury prevention in a variety of sports have found substantial decreases in injuries (up to 72%) [13–17], a recent study attempting to translate an evidence-based warm-up to a high school athlete population did not demonstrate a reduction in lower extremity injuries, most likely due to poor adoption and adherence (32%) [5]. Additional research is needed to elucidate the barriers and facilitators to the implementation, adoption, and sustained use of proven warm-up routines among high school athletes.

Our study aims to inform the design of an injury prevention warm-up strategy and to increase the likelihood that it is adopted and adhered to by high school basketball teams. We conducted semi-structured interviews with high school basketball coaches and focus groups with players to describe the current state of warm-up programs in the high school basketball arena and to better understand what factors will influence adoption and adherence. We also assess the coaches’ and players’ beliefs in existing evidence, adherence to standard warm-up practices, and perceived barriers and facilitators to consistent engagement in evidence-based warm-up programs.

## Methods

We employed qualitative research methods as part of an exploratory study of high school basketball coaches and players from a school district in Southern California from May 2018 to October 2019. Our partner district has 8 comprehensive high schools with just over 25,000 students enrolled during the 2018–2019 school year, an ethnic diversity index of 39/100, and just under 15,000 students eligible for free and reduced price meals. The district contains a combination of highly competitive top-tier basketball teams and smaller developing high school basketball programs for both boys and girls. All 16 high school basketball head coaches in the district were invited; 12 coaches (75%) participated in the study, and 4 never responded to our recruitment email and phone call attempts. Coaches were asked to assist with recruiting their own players (e.g., 6–8 players per team) for focus groups. Having interviewed the majority of coaches in the district (12 of 16), we were confident data saturation was achieved with these stakeholders; with the player focus groups, we stopped initiating additional interviews when no new themes were emerging from these discussions [18–20]. We completed a total of five player focus groups representing both boy and girl teams, and 5 high school campuses.

### Data Collection Procedures

Interviews and focus groups were conducted by experienced qualitative researchers on the study team. Semi-structured (open-ended) interview and focus group guides facilitated the discussion with coaches and players (see Interview Guides, Supplemental Digital Content 1). The content of the interviews for the coaches includes five primary domains of questions: (1) current practice; (2) knowledge re: evidence of effective warm-up strategies; (3) beliefs; (4) barriers and facilitators; and (5) team injury experience. The content of the interviews for the players included (1) current practice, (2) beliefs, and (3) barriers and facilitators. Coach interviews lasted approximately 30–60 min; player focus groups were scheduled for 90-min sessions. Remuneration was provided (e.g., coaches received $100 gift cards; players received “swag” bags containing sports-related gear worth approximately $25 per player). All discussions were audio-recorded with the permission of the participant(s). The digital recordings were transcribed by an approved vendor providing transcription services to the Department of Research and Evaluation at Kaiser Permanente Southern California.

### Data Coding and Analysis

We employed thematic analysis of the qualitative data using team coding and the constant comparative method, allowing the study team to establish coder agreement critical for “trustworthiness” in qualitative research [21–26]. A lead and secondary coder reviewed and independently coded a sub-sample (*n* = 3) of transcripts. Coding categories were initially derived deductively from a priori research questions outlined in the semi-structured interview guides. Additional coding categories were inductively derived as the coding team identified new and unexpected themes grounded in the data while actively coding. Once the selected transcripts were independently coded, both coders met repeatedly; these meetings were critical to the analysis, as disagreements between coders were reviewed and discussed until consensus was achieved and the coding structure revised. To further ensure rigor in the process, codes were documented in a finalized codebook, and the lead coder applied the consensus-based codes to the remaining transcripts (*n* = 14) [24]. Once coding was complete, both coders met again to interpret the thematic findings and agree on a thematic hierarchy for summary purposes.

### Study Population

We interviewed 12 varsity basketball coaches individually (9 male; 3 female) and 30 high school basketball players as part of five focus groups (player characteristics are presented in Table 1).

**Table 1:** Characteristics of high school basketball players participating in focus group interviews (*n* = 30)

	Male	Female
Age		
14 years old	0	1
15 years old	2	2
16 years old	2	11
17 years old	6	5
18 years old	1	0
Gender	11	19
Ethnicity		
Asian	1	1
Black/African American	4	2
Hispanic/Latinx	3	13
White	2	1
Others	1	2

## Results

We present the thematic results from the interviews and focus groups below, including coaches’ and players’ experience with warm-up routines, knowledge, and beliefs about warm-up programs designed to reduce injury, and environmental-, player- and coach-level barriers to participation and engagement in these types of activities.

### Current Engagement of Teams in Routine Warm-Up Programs

Coaches and players reported engaging in warm-up exercises regularly (e.g., daily), most often as a formal part of practice. Teams spent a range of 5–45 min warming up (mean = 17 min; mode = 15 min) during practice, but less time at games and during the off-season. Most teams performed the same warm-up routine consistently. The majority of coaches relied on team captains or players to lead/co-lead the warm-up*.*

In addition to the variability in time spent on warm-up activities, the content, format, order, and structure of the warm-up programs varied across teams. Despite this variation, teams generally incorporated light jogging and/or jump rope with some combination of static stretching (i.e., toe touches, ankle stretches), dynamic stretching (i.e., karaokes, high kicks, lumberjacks, rear kicks, lunges), and drill-based exercises (i.e., full or half court layup lines or other ball drills) into their regular routines. While some coaches believed there was room for improvement in their warm-up programs (*There is a proper way [to warm-up], and I know we are probably not doing it.*), participants perceived their current warm-up routine as a good start.

### Knowledge and Beliefs Regarding the Value of Warm-Up Routines to Prevent LEIs

Coaches and players reported awareness and consensus that warming-up prevents injuries. Echoing other coaches, one individual commented, “I am not as highly educated to say I understand exactly how every stretch is going to protect us, but I do understand that warming yourself up and stretching…is a way to protect you from injury.”

Coaches often relied on experience rather than knowledge of the current research to inform their warm-up routines. While coaches do not rely heavily on outside recommendations for warm-up guidance and do not have extensive knowledge of the current evidence, they did cite a few sources of information, including (1) school district materials required for coaching certification, (2) information provided by players’ parents, (3) coaching magazines, (4) observation (e.g., local college practices), and (5) guidance from colleagues (professional strength and/or conditioning coaches).

Although coaches expressed varying perspectives on the best approach to warming-up (i.e., types of exercises, sequencing, time spent), participants want to learn more about the evidence-based warm-up practices that are most effective for preventing lower extremity injuries (“There are things that I would like to do, if I knew techniques to use, that I would incorporate.”).

### Barriers and Facilitators to Engagement in Routine Warm-Up Programs

Identified barriers to current warm-up engagement include environmental (access to gym space), player-level (e.g., players’ sense of invincibility, not taking warm-up seriously), and coach-level challenges (e.g., competing priorities and a lack of knowledge about evidence-based warm-up). Facilitating factors include player and team injury experience and coach engagement. Table 2 provides a summary of identified barriers and facilitators with representative quotes.

**Table 2:** High school basketball coaches’ and players’ perspectives of barriers and facilitators to warm-up at practice and games

**Barrier level**	**Barrier**	**Representative quotes**
Environmental	Access to gym space/limited time in the gym	“If we are on the road, we don’t always have access to a gym or as much time; we may be waiting for a [Junior Varsity] game to finish.” [coach] “I mean, in the offseason…it’s great that volleyball lets us have that one hour or whatever, but then they’re coming in [to the gym], and we got to get out…it’s not even an hour…I had even mentioned to our [athletic director]…’Man, if we can back volleyball off to 3:15,’ I’m like, ‘That 20 minutes would make a huge difference.” [coach] “So, after the freshman game, then JV plays and then we play, so it’s not like we’re in two different gyms. We’re in the same gym, so we have to wait for their game. I think that’s what affects our warmup time…Because they want to keep everything on schedule.” [player] “Yeah, I think, if I had more time, I think I’d probably lend myself more to exercises, and stretches, and cooldown things at the end. Like I said, a lot of times, you’ll get going into practice, and then, you know, you’re losing track of time. Before you know it, your time is up. The other team is coming in, and you got to get off the floor.” [coach] “And we don’t have that much space to stretch sometimes.” [player]
Player level	Player invincibility	“…the bottom line is [our players] don’t always take their stretching very seriously…They don’t understand the importance of it as far as helping them to prevent injuries…they think they’re invincible.” [coach] “Because sometimes, no matter what you say to kids, they don’t really understand it until it happens to them. And then they’re like, yeah, I do need to – you know, do my ankle exercises. I do need to drink water. Because they think they’re invincible…” [coach]
	Not taking warm-up seriously	“…some of the kids don’t see the benefit [of warming up] and sometimes they won’t listen.” [coach] “But I feel like when…we get [warm-up] done fast, it’s because the girls are serious, and they actually want to get it done. And then when [it takes longer]…it’s because girls are messing around and they’re not taking it serious.” [player] “I think the team not focusing, a lot of people messing around and stuff [is a challenge]…So, then not really stretching and we’re joking around, makes it hard to focus on the game…warming up and stuff.” [player]
Coach level	Competing priorities	“The one thing that we’re going to cut out, if we have a game the next day…is going to be a little bit of the stretches…But we still think that it’s important and we’re trying to figure out how we can incorporate it to make it important every single day.” [coach] “…sometimes time is an issue….this time of our year, at the beginning when we only have 50-55 minutes…spending ten minutes of that…to [warm-up]…I haven’t placed enough priority on doing it…” [coach] “…warmup is probably the last thing…I think about in my thought of what we need to do.” [coach] “…the trade-off is always, okay, we have…all these things on my practice plan…we hope they’re mentally prepared but we don’t always make sure that they’re physically…ready to go…” [coach]
	Lack of knowledge	“I don’t think I do a good enough job of explaining exactly what we’re stretching correctly, of demonstrating…what we’re doing – [the] correct form and all that.” [coach] “But, yeah, if there’s something that I could learn…[that] is time effective and would fit in, I would love to…have it…” [coach] “…everyone has their own opinion on warmups…We all have our own way of doing it, so there’s not like a set thing that we really know works.” [coach] “Again, right now – because I’m so limited in knowledge on it – I would revert back to what we’ve been doing.” [coach] “Now, I will say there are – like I think what I feel like I’m lacking in my warmup…is basketball is a tremendous amount of load on your knees, ankles, back, and hips. It’s unlike any other…So, there are things that we don’t do. Like, okay, so if you’re a basketball player and you’re in…an extreme athletic position, where so much load is on your thighs, your glutes, and your hips. The things I just…described – how much of that is really working on those things?...I have often thought…we need to do more like lateral, defensive-mimicking, dynamic movements to warm up.” [coach]
**Facilitator level**	**Facilitator**	**Representative quotes**
Player level	Past injury experience	“And sometimes, it takes them….I don’t want to say [to] get injured. But to kind of go through a situation where they might have a little bit of an injury and then they learn the importance of recovery. And prevention. Because sometimes, no matter what you say to kids, they don’t really understand it until it happens to them. And then they’re like, ‘Yeah, I do need to…my ankle exercises. I do need to drink water.” [coach] “[I encourage other players to]…take it seriously…I got hurt from not stretching properly. So, I don’t want them to go through what I went through, not being able to play. Because it’s just like sitting on the sidelines and watching everyone else play…I was out like six weeks.” [player]
Coach level	Coach engagement	“[Coach] puts a big emphasis on it…He makes sure we do it right…you have the right form…Plays close attention to the little details, too…Our coach usually gives a really good explanation, like, “Oh, yeah, this is what it really does,” and what’s it’s working out, and this, or it doesn’t do this or this. He usually explains why we’re doing everything.” [player] “I didn’t care that much…because my [prior] coach didn’t make it seem like a big deal, so I was like, “Why should I care if she doesn’t?”…And then I would, after games, I’d feel very sore in my body and everything felt tight, and it’s because I wouldn’t stretch. And I didn’t realize that because no one told me, and then [Coach X] pretty much, because he put so much emphasis on it, I was like, it’s important.” [player] “…I also feel like it’s mostly the coaches, because they’re the ones who implement the time structures to the practice, like, “We’re doing this for this amount of time.” So, it’s like, if they give us the time to do it, we’re going to do it.” [player]

#### Environmental-Level Barriers

Coaches and players acknowledged their current warm-up routines suffer at games, citing both a lack of time and constrained space as primary challenges. Space constraints at games often result in players foregoing dynamic stretches for static ones “…because you don’t have room to really do movement sort of things.” While games present a bigger challenge, access to gym space can also be an issue for practice because multiple teams often share space.

#### Player-Level Barriers

Some players are described as maintaining attitudes that disrupt their engagement in warm-up, including their internalized sense of invincibility. Younger players are perceived as particularly lacking a clear understanding of the importance of warming up to prevent injury (“I think with the young kids…they think, ‘oh I can’t get hurt’…they don’t take it seriously.”).

#### Coach-Level Barriers

Coaches struggle with competing priorities when it comes to managing their teams at both practice and games, admitting warm-up activities often take a back seat to other tasks in their practice plans. Some coaches monitor to ensure players are engaging in the warm-ups, even if they rely on players to lead the warm-up (“I certainly am prompting everything they’re doing…”)*.* Other coaches, however, experience distractions during the team’s warm-up period preparing for practice and coordinating with their assistant coaches (“I’m going to be honest. We’re not monitoring [players] that much during [their warm-up]…as coaches [we are]…debriefing, getting ready for practice….we’re going to go over X, Y, and Z.”). One participant warned, “ If coaches aren’t observant…[and] you walk away…[players] won’t do [the warm-up]….”

Finally, coaches reported a lack of knowledge and confidence about their abilities to (a) select the appropriate exercises to target the prevention of specific injuries, (b) properly sequence exercises during warm-up to prevent injury, and (c) teach their players proper stretching form. Players echoed their coaches’ concerns, desiring more information about specific stretches to prevent particular injuries (“Personally, I’d be like, “What’s the best stretch for your knees?” Because I do jump a lot.”) and more guidance on proper form (“Make sure you know all the stretches correctly and how to do it…just to show us the right way…”).

Coaches and players also identified facilitators to current warm-up engagement include player level (past injury experience) and coach level (coach involvement and player encouragement).

#### Player-Level Facilitator

Players’ previous injury experience shaped their perceptions and attitudes about warming up to prevent injury (“An injury [gets players to understand warming-up is important]…Yeah [laughter]. I’m sorry…I know it sounds terrible…I don’t wish an injury [on anyone]…”). Many players have participated in sports since they were in elementary and junior high school, admitting that they did not take warming up seriously when they were younger (“I’d just put on my shoes and I’d start playing…[until]…My knee [injury].”). Describing when that personal belief changed, another player clarified:

[I started taking warm-up seriously in] eighth grade… tweaked my knee and I had growing pains. That was it…Just having to sit out, it’s just like, “I could have prevented that by actually stretching.”…[before that I thought] “We’re fine. We’re young.” We can run around and be fine.

#### Coach-Level Facilitators

In addition, coach involvement and encouragement are key factors that facilitate getting players to prioritize warming up. For instance, one coach motivates younger players through a practice of providing, “…recognition for [warming up and] a reason why this is good. And so, it motivates them. ‘Okay, so, coach notices. Coach sees it. So, let me do it…’”. When their coach emphasizes the value of warm-up it keeps players “…on task rather than just like, if we’re doing it on our own, I feel like we can probably mess around with each other and not focus on the stretching. Rather when there’s discipline, we have to do it…”

## Discussion

Our results help to better understand the warm-up practices of high school basketball coaches and players, as well as their knowledge and attitudes about evidence-based strategies for preventing lower extremity injuries. We found that regular involvement in basketball warm-ups was common among high school teams, but the methods and time dedicated to these practices varied substantially despite teams belonging to the same school district.

Players and coaches are eager for more information on warm-up programs proven to reduce lower extremity injuries, as well as recommendations for effective implementation. Our finding that coaches and players are aware of the preventive value of warm-up routines and express a willingness to use an evidence-based warm-up routine has been demonstrated in previous research [27]. However, our stakeholders also identified multiple barriers to adherence. Similar to prior research, we found access to gym space before practice and games to be a challenge [5]. Our interviews also highlighted the presence of competing demands during limited practice time. These findings may suggest the desirability of a warm-up program that is relatively short in duration. Additionally, younger players have a sense of invincibility and held the belief that they should focus on specific stretches that would prevent very targeted injuries (rather than warming up generally). Finally, coaches report a lack of knowledge regarding the design and instruction of evidence-based warm-ups.

**Fig. 1:**
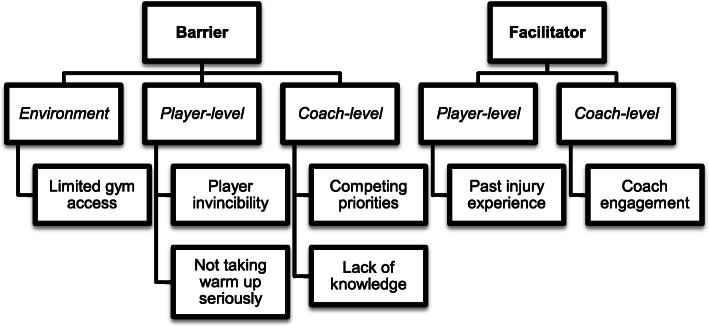
Coach and player identified barriers and facilitators to engaging in routine warm-up to prevent lower extremity injuries

We also identified factors that facilitate consistent adherence to warm-ups. In our study, many of the players reported at least one previous injury. Some injuries could be categorized as overuse injuries, while many were serious acute injuries requiring significant physical therapy and/or surgery. Players explained that previous injuries facilitated a deeper engagement in warm-up routines to prevent additional injury. In addition, players and coaches both endorsed that coach attention and recognition of players’ warm-up activities were motivational. Figure 1 provides a visual summary of the emergent barriers and facilitators.

The PRECEDE-PROCEED model is an eight-part framework used to provide a standard structure for designing, implementing, and evaluating public health programs [28–34]. The PRECEDE stage of the model includes four phases of intervention planning: (1) social diagnosis; (2) epidemiological, behavioral, and environmental diagnosis; (3) educational and ecological diagnosis; and (4) administrative and policy diagnosis. The PROCEED stage includes four additional phases: (5) an implementation phase, (6) process evaluation, (7) impact evaluation, and (8) outcome evaluation. In the context of this model, our findings could be useful to the design of an intervention to decrease lower extremity injuries for high school basketball players through adoption and adherence to a specific warm-up or training program.

Viewing our findings through the PRECEDE phases of this model, we reflected that the high level of engagement in our study by district and school officials was reflective of interest in preventing lower extremity injuries (social diagnosis phase). Coaches cited the desire for knowledge around effective warm-up strategies reflects a readiness to change warm-up practices.

Behavioral and environmental factors relevant to the design of a warm-up intervention included distractions and competing demands on coaches’ attention, delegation of warm-up oversight to players, and availability of other resources, such as equipment. The lack of access to gym space and adequate time in the gym at both practice and games are external conditions that can hinder players from engaging in a consistent and adequate warm-up (“Sometimes, we feel like we’re rushed because there could be a game going on…”).

In the domain of educational and ecological factors, we observed an array of *predisposing*, *enabling*, and *reinforcing factors* that can be leveraged in the design of a warm-up intervention. One of the strongest predisposing factors was players’ and coaches’ belief that an evidence-based warm-up program can effectively prevent lower extremity injuries. Furthermore, while the high school athletes we interviewed highlighted the benefits of warm-up, they perceived little to no risk associated with this activity. For many of these athletes, their predisposed belief in the effectiveness of warming-up to prevent/reduce injury was grounded in their own injury experience or witnessing teammates’ injuries (“An injury [gets players to understand warming-up is important]…I’m sorry…I know it sounds terrible…”).

Despite the variability in the types of warm-up exercises conducted across teams, our finding that all teams routinely incorporate time for warm-up into their practice plan can be viewed as an enabling factor, in that it provides the opportunity for players to adopt and maintain participation in these activities. Coaches also cited some techniques for obtaining information about effective warm-ups, such as consulting with colleagues, observing local elite teams, and reading coaching magazines or certification materials. However, coaches agreed that standard guidance for effective warm-ups was not readily available to them, which is an opportunity to better enable effective warm-ups. Coaches’ attempts to get players to buy-in and engage in warm-up activities serve as a reinforcing factor, as players acknowledged that they respond to this type of encouragement and demonstrated discipline (“…you think [warming-up is] more important because [the coach is] putting so much emphasis on it…it just makes you want to do it more, because he also does it….he really believes in it…”). Coaches further noted that explaining the reasons for warming up was reinforcing factors strengthening player motivation. Some players also provided reinforcing support by encouraging their peers to take warm-ups seriously, following their own injuries. These sorts of peer and social supports may be key to increasing the adoption of effective warm-up routines.

### Limitations

This study used exploratory qualitative research methods to elicit participants’ perspectives. Consistent with this research approach, our study represents a small, but adequate sample size. While the purposive sampling methods employed may not be generalizable to high school basketball players and coaches across all settings, our findings offer rich, context-specific insights into the experiences and perspectives of our participants. We used standard and structured coding methods to extract themes from the raw data and employed independent coders for analytic rigor. While we acknowledge participant and researcher biases can be a limitation in qualitative studies, we tried to mitigate this form of bias by employing team coding (the use of multiple coders) and assessing when we believed we had reached data saturation (e.g., when we started hearing the same themes over and over again across our interviews). In addition, our total number of participants (*n* = 42) in this study actually represents a reasonably large sample size for an exploratory qualitative study. Ultimately, our results contribute to the limited prior studies published in this area of research in the current literature.

## Conclusions

Researchers, sports-medicine professionals, coaches, and other stakeholders interested in developing and implementing effective warm-up routines should consider identified barriers and facilitators to inform future interventions targeting high school basketball players and teams. Further exploration is needed to better assess how these challenges may impact compliance with effective warm-up practices, since adherence to a proven warm-up program will be a key determinant of reaching desired outcomes.

## Supplementary Information

**Additional file 1: Supplemental Digital Content 1.** Coach and Player Interview Guides.

## Data Availability

Due to the nature of this research, participants of this study did not agree for their data to be shared publicly, so supporting data is not available.

## Abbreviations

MPH: Master’s Degree in Public Health
MS: Master’s degree in science
PhD: Doctor of Philosophy
MD: Medical doctor
MSc: Master’s degree in science
CA: California
LEIs: Lower extremity injuries
KP: Kaiser Permanente
