# Supplementary material for: High School Basketball Coach and Player Perspectives on Warm-Up Routines and Lower Extremity Injuries
Source: Sports Med Open. 2021 May 21;7:34. doi: 10.1186/s40798-021-00328-4 (PMC8140014; doi:10.1186/s40798-021-00328-4)
Supplement: Supplementary file 1 — Additional file 1: Supplemental Digital Content 1. Coach and Player Interview Guides. [file 40798_2021_328_MOESM1_ESM.docx]

**Supplementary Digital Content 1.doc**

**Semi-Structured Interview Guide: Coaches**

**Section 1: Current Practice**

***We would like to begin by asking a few questions about your current approach to warm-up activities.***

1. What, if any, warm-up activities do your players regularly engage in before practice/games?

Probes:

- 1. Are warm-up activities typically a team activity, or are players expected to do them on their own?
  2. Before or during practice?
  3. Structured vs. unstructured approach?
  4. Time allotted?
  5. Who leads the warm-up?
  6. How consistently do players/the team warm up?
  7. Does anyone track that warm-ups are being done? If so, how? Who?
  8. Routine different at practice vs. games?
  9. What if a player shows up late to practice, what happens then?

1. What specific types of warm-up exercises or routines do your players do at practice?

Probes:

- 1. How variable is your warm-up routine? Are there certain exercises your team always does?
  2. Why are these types of exercises performed?
  3. Why do you think these types of exercises are important for warm-up?
  4. Are there other types of warm-up exercises you would like to see your players do? If so, what types of exercises?

1. How do you approach talking to your players about warming up?

Probes:

- 1. What information do you share to encourage them to warm-up?
  2. What strategies do you employ to get your players to follow through on warm-up activities? Incentives?

**Section 2: Knowledge re: Evidence of Effective Warm-up Strategies**

***We are also curious about any evidence you may be aware of and/or rely on for guidance on effective warm-up routines for your team.***

1. What, if any, professional guidelines or recommendations are you aware of about effective warm-up routines for your players?

Probes:

- 1. Specific source of information?
  2. Belief in the quality of the evidence (or go more off their own experience)?

**Section 3: Beliefs**

***Thank you for your answers so far, they are extremely helpful. My next questions are about the relative importance you place on warm-up activities overall.***

1. How much of a priority do you place on encouraging your players to engage in warm-up activities?
2. To what extent do you believe an evidence-based warm-up routine can be effective in preventing lower-extremity injuries among your players?

**Section 4: Barriers & Facilitators**

***Let’s spend the next few minutes talking about any challenges you may experience implementing warm-up into your team’s routine, and suggestions for overcoming those challenges.***

1. In your experience, what makes it difficult to implement an effective warm-up routine with your team?

Probe:

- 1. What strategies, if any, have you used to overcome these challenges?

**Section 5: Team Injury Experience**

1. To what extent have lower-extremity injuries been a problem for your current roster of players?

Probes (if injuries cited):

- 1. How have those injuries impacted the specific player(s)? The team?

1. Are your players’ injuries tracked in any way?

Probes (If so):

- 1. How are injuries tracked? Who tracks them?

Probes (if not):

- 1. Do you track bench time or absence due to injury, even if you don’t record the injury itself?
  2. (If not): What do you think would be the best way to track player injuries? / would it be feasible to track injuries or absence/non-play due to injuries?

**End of Interview Questions**

***That concludes my questions for today. Do you have any final thoughts or suggestions?***

***Again, I want to thank you very much for taking the time to participate in this interview***.

**Semi-Structured Focus Group Guide: Players**

**Section 1: Current Practice**

***Let’s begin by discussing what your team typically does to warm up, if anything.***

1. What, if any, warm-up activities do you do at practice or games?

Probes:

- 1. Are warm-up activities typically a team activity, or are you expected to do them on your own?
  2. Before or during practice?
  3. Structured vs. unstructured approach?
  4. Time allotted?
  5. Who leads the warm-up?
  6. Routine different at practice vs. games?
  7. What if a player shows up late to practice, what happens then?

1. In what ways does your coach encourage you to do warm-up exercises before practice/games?

Probes:

- 1. How much does your coach prioritize warm-up activities?
  2. To what extent have you and your coach(es) discussed warm-up exercises or routines? What did you specifically talk about?
  3. Do you feel that you get enough time to warm-up effectively at practice/games?

1. What specific types of warm-up exercises do you do?

Probes:

- 1. How did you learn about these particular exercises?
  2. Which of these exercises are your favorite?
  3. Are there other types of warm-up exercises you would like to engage in at practice/games that you don’t currently do? If so, please describe.

**Section 2: Beliefs**

***Thank you, that really helps us to understand what your team currently does for warm-up activities. Now I want to ask you about how important you personally think it is to regularly warm-up.***

1. In your opinion, how important do you think it is to warm-up at practice/games?

Probes:

- 1. As an athlete, what benefits do you hope to get from warming-up?
  2. Alternatively, do you have any concerns about stretching (e.g., injury)?

1. What do you consider as warm up (i.e., static stretching, drills with the ball, both, other?)

**Section 3: Barriers and Facilitators**

***Let’s now take a few minutes to talk about what, if anything, gets in your way of regularly warming- up at practice/games.***

1. What makes it easy for you to regularly engage in a warm-up routine?
2. Alternatively, what makes it difficult for you to regularly engage in a warm-up routine?

**Section 4: Injury Experience**

1. Have you ever had a sports related injury?

Probes **(if so)**:

- 1. Please describe the injury. How did the injury occur?
  2. How did the injury impact you?
  3. Have you fully recovered from that injury?
  4. Are you concerned about re-injury? Strategies to avoid?
  5. **(If no)**: Why do you think you’ve never had an injury?

**End of Focus Group Questions**

***That concludes our questions for today. Are there any final thoughts or suggestions?***

***Again, I want to thank you very much for taking the time to participate in this focus group discussion***.
